# Gammaherpesvirus Latency Accentuates EAE Pathogenesis: Relevance to Epstein-Barr Virus and Multiple Sclerosis

**DOI:** 10.1371/journal.ppat.1002715

**Published:** 2012-05-17

**Authors:** Costanza Casiraghi, Iryna Shanina, Sehyun Cho, Michael L. Freeman, Marcia A. Blackman, Marc S. Horwitz

**Affiliations:** 1 Department of Microbiology and Immunology, The University of British Columbia, Vancouver, British Columbia, Canada; 2 Trudeau Institute, Saranac Lake, New York, United States of America; Emory University, United States of America

## Abstract

Epstein-Barr virus (EBV) has been identified as a putative environmental trigger of multiple sclerosis (MS), yet EBV's role in MS remains elusive. We utilized murine gamma herpesvirus 68 (γHV-68), the murine homolog to EBV, to examine how infection by a virus like EBV could enhance CNS autoimmunity. Mice latently infected with γHV-68 developed more severe EAE including heightened paralysis and mortality. Similar to MS, γHV-68EAE mice developed lesions composed of CD4 and CD8 T cells, macrophages and loss of myelin in the brain and spinal cord. Further, T cells from the CNS of γHV-68 EAE mice were primarily Th1, producing heightened levels of IFN-γ and T-bet accompanied by IL-17 suppression, whereas a Th17 response was observed in uninfected EAE mice. Clearly, γHV-68 latency polarizes the adaptive immune response, directs a heightened CNS pathology following EAE induction reminiscent of human MS and portrays a novel mechanism by which EBV likely influences MS and other autoimmune diseases.

## Author Summary

Multiple sclerosis (MS) is a chronic inflammatory disease of the central nervous system (CNS) that leads to progressive disability. The causes of the disease are still unknown. Viral infections has been linked to MS development and Epstein-Barr virus has been shown to have a strong link to MS. Here, we use an animal model of MS, experimental autoimmune encephalitis (EAE), to study how EBV can trigger MS. Since EBV does not infect rodents, we infected mice with murine gamma herpesvirus 68 (γHV-68), the murine equivalent of EBV. We found that mice previously infected with γHV-68 developed more severe EAE when compared to uninfected EAE mice and showed pathological features that recapitulate human MS. γHV-68 EAE presented with increased CD4 and CD8 T cells infiltrations in the brain, increased brain inflammation and demyelination. This model provides new insight on how EBV might be triggering MS, shedding light on the development of the disease.

## Introduction

Multiple sclerosis (MS) is a chronic inflammatory disease of the central nervous system (CNS). In MS, the myelin sheath, that insulates and protects neurons, is attacked and destroyed by the host's immune system leading to progressive disability [1]. MS is considered to be the result of an environmental influence in genetically susceptible individuals. Different environmental triggers have been associated with MS development and viral infections are the most common suspects [1]–[2].

To date, Epstein-Barr virus (EBV) has the strongest correlation with MS development [2]. EBV is a DNA virus of the γ-herpesvirus family. It establishes a life-long latent infection mainly in B cells [3], but it has been shown to have the ability to infect other cell types such as epithelial cells, endothelial cells and monocytes [4]–[5]. It infects 90% of the human population and it is usually acquired during childhood. When primary infection takes place during adolescence or in adulthood, it leads to infectious mononucleosis [6]. All MS patients are EBV seropositive [7] and individuals with a history of mononucleosis have a 20-fold higher risk of developing MS later in life [2]. Moreover the EBV-specific humoral and cellular immunity seems dysregulated in MS patients: there is an increase in anti-EBV antibodies titer years before MS onset [8], a decreased CD8+ EBV response [9] and the presence of polyfunctional myelin/EBV cross-reactive CD4+ T cells in MS patients [10]. Finally, although still controversial, EBV infected B cells have been found in ectopic follicles in the brain of MS patients [11]–[14]. Despite all this evidence, it is still not clear how EBV would trigger CNS autoimmunity. Although EBV does not infect rodents, murine gamma herpesvirus-68 (γHV-68) has been a useful tool in studying the relationship between the immune system and latent γ-herpesvirus infection in mice [15].

Experimental autoimmune encephalomyelitis (EAE) is a well-studied and accepted model for the study of MS in rodents [16]. After immunization with myelin peptides emulsified with adjuvants, mice develop ascending paralysis and present with CD4 T cell and macrophage infiltrations in the white matter of the spinal cord, with minimal brain inflammation. In MS, however, the vast majority of myelin lesions are found within the brain parenchyma and equivalent numbers of CD8 T cells are found alongside with CD4 T cells, both playing critical roles in the disease pathology [1], [17]. Despite these differences and others, EAE has proven to be a valuable tool in the development of therapies that are now being successfully used to treat MS [18].

Although γHV-68's genome differs from EBV, it elicits an immune response in mice that shares many features with EBV. In fact both viruses establish a life long infection in B cells, deeply modulating the immune response of the host, leading to the expansion of a potent CD8 response similar to that detected in humans during mononucleosis [19]. Because of these similarities, we decided to test the impact of a γHV-68 latent infection during the development of EAE. When induced for EAE, mice latently infected with γHV-68 showed a significantly modified disease phenotype that recapitulated aspects of human MS not typically observed in EAE. γHV-68 EAE mice presented with greater ascending paralysis, more neurological symptoms, brain inflammation with myelin lesions driven by a potent Th1 response and CD8 T cell infiltrations.

## Results

### Latent infection with γHV-68 enhances EAE symptoms without CNS infection or increased viral reactivation

**Figure 1:**
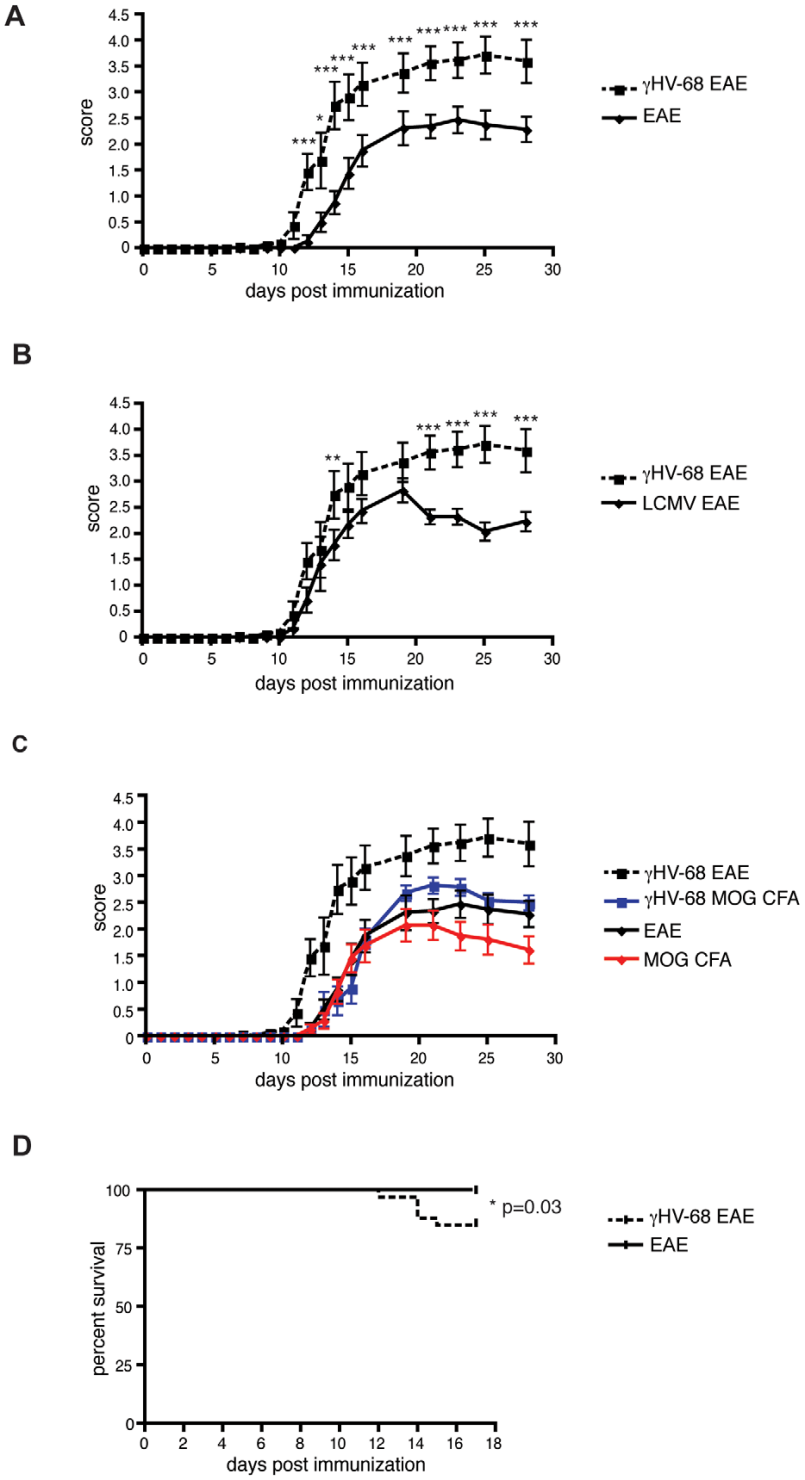
γHV-68 infected mice show worse EAE symptoms and increased mortality. Mice were infected with γHV-68 (dashed line) or LCMV or MEM only (solid lines). Five weeks p.i. EAE was induced (day 0 on the graph) with (EAE) or without (MOG CFA) pertussis toxin. (**A-B-C**) EAE scores up to day 28 post EAE induction (three separate experiments, n = 16/group). Data are shown as mean score. Data analyzed with two-way ANOVA followed by Bonferroni's post test; *** p<0.001; ** p<0.01 * p<0.05. (**D**) Survival up to day 18 post EAE induction (four-five separate experiments, n = 28/group). Data analyzed with Kaplan-Meier analysis.

To determine the impact of γHV-68 latency on the development of EAE in mice, C57Bl/6 mice were infected with γHV-68 and allowed to recover for five weeks to enable complete clearance of the acute infection and establishment of latency prior to EAE induction. Viral clearance was demonstrated by absence of plaques on viral plaque assays on spleens harvested on day 35 post infection. When tested, mice previously infected with γHV-68 presented with earlier onset of EAE (around day 7–8 post induction), compared with naïve mice induced for EAE that developed paralysis around day 10–12 post EAE induction (Figure 1A). Moreover γHV-68 EAE mice presented with worse clinical EAE scores (Figure 1A) and this includes a heightened mortality rate (15%) between day 12–18 post EAE induction (Figure 1D). To demonstrate the specificity of the disease phenotype to γHV-68 infection, EAE was induced in mice previously infected with Lymphocytic Choriomeningitis virus (LCMV). LCMV induces a strong well-characterized Th1 response as well as a strong memory CD8 T cell response similar to γHV-68. Mice previously infected with LCMV (LCMV EAE), showed a clinical disease course similar to uninfected EAE mice (Figure 1B). At the dose given, LCMV does not establish a latent or persistent infection, so to further test the γHV-68 specificity of our phenotype, mice were infected with a β-herpesvirus (murine cytomegalovirus, MCMV), capable of establishing a latent infection similar to γHV-68. MCMV EAE mice developed EAE with the same clinical disease course of uninfected EAE mice and showed 100% survival after immunization (data not shown). Additionally, EAE was induced in γHV-68 mice without administering pertussis toxin (γHV-68 MOG CFA). Interestingly, γHV-68 MOG CFA mice (Figure 1C, blue line) developed milder paralysis than γHV-68 EAE mice, yet still displayed a disease course similar to EAE alone (Figure 1C, black line). These data clearly demonstrate that this phenotype is a feature specific for γHV-68 latent infection as only latent γHV-68 infection confers susceptibility to a more severe form of EAE that includes a heightened level of mortality.

**Figure 2:**
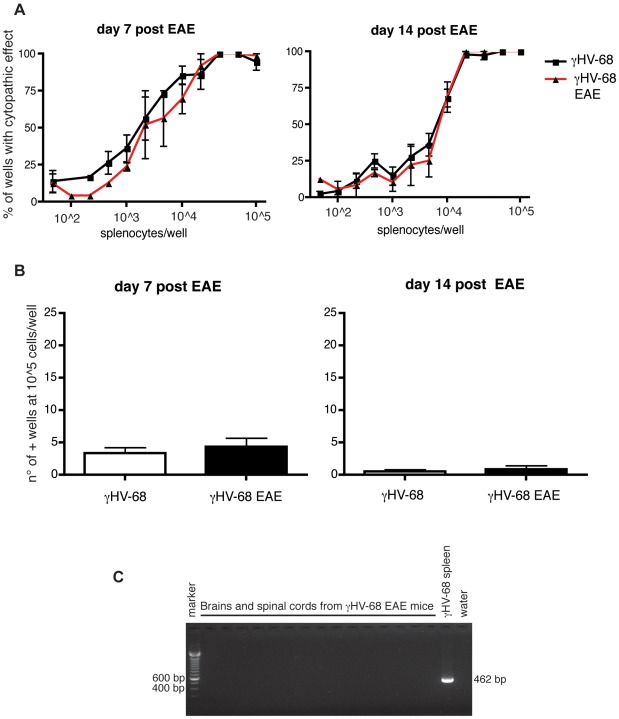
γHV-68 does not reactivate more upon EAE induction and does not infect the CNS. Mice were infected with γHV-68. Five weeks p.i., EAE was induced (red line with triangles, black bar) or not induced (black line with squares, open bar). At day 7 (left panel) and day 14 (right panel) post EAE induction, spleens were harvested and a limiting dilution assay was performed using (**A**) live splenocytes to assess the amount of ex-vivo viral reactivation and (**B**) lysed splenocytes to assess the amount of pre-formed virus (three separate experiments using 3–4 mice/group) (**C**) Brain and spinal cords were harvested from perfused γHV-68 EAE mice at different timepoints pre and post EAE (day 0 before EAE induction; day 3; day 7 and day 14 post EAE). DNA was extracted and a nested PCR was performed to detect γHV-68 DNA. A spleen was used as a positive control. Figure C shows a representative gel of three separate experiments (15 brains/spinal cords harvested at day 14 post EAE induction have been loaded on the gel displayed here; 37 brains and 39 spinal cords were analyzed in total).

Previous work has demonstrated that reactivation of γHV-68 in latently infected mice occurs following treatment with toll like receptors (TLR) ligands such as poly I∶C or LPS [20]. For EAE induction, mice are injected with complete Freund's adjuvant (CFA) and pertussis toxin (PTX) which are constituted with TLR ligands. To determine whether the increased EAE symptoms observed were due to increased viral reactivation and replication, limiting dilution assays were performed to allow for the simultaneous quantification of ex-vivo γHV-68 reactivation (Figure 2A) and pre-formed virus (Figure 2B). γHV-68 EAE mice showed a similar extent of ex-vivo reactivation and similar amount of pre-formed virus in the spleen both at day 7 and day 14 post EAE induction when compared to γHV-68 infected mice (5–7 weeks post infection) without EAE induction. TLR ligands present in the CFA are not reactivating more γHV-68 and enhanced EAE scores are not due to increased viral replication.

To further confirm the lack of dependence on viral replication/reactivation, EAE was also induced in uninfected IL-6KO mice and in γHV-68 IL-6KO mice. IL-6KO mice are resistant to EAE induction and both latently infected and uninfected mice retained this resistance. Moreover, γHV-68 mice were treated with cidofovir, a drug known to suppress γHV-68 replication [21], before and after EAE induction. γHV-68 mice treated with cidofovir showed no differences in phenotype and clinical disease as compared to untreated γHV-68 mice (data not shown).

Finally, to ask whether virus replication was detectable in the CNS, DNA was extracted from brains and spinal cords and the presence of viral DNA was assessed using the most sensitive PCR approach (nested PCR; Figure 2C). All brains and spinal cords tested negative for γHV-68 DNA following EAE induction. Overall these results indicate that the observed phenotype was not the result of a reactivated replicating virus and that latent γHV-68 infection is inducing greater disease through an indirect mechanism.

### γHV-68 EAE mice have increased CD8/CD4 T cells infiltrations, increased inflammation and MS-like lesions in the brain

**Figure 3:**
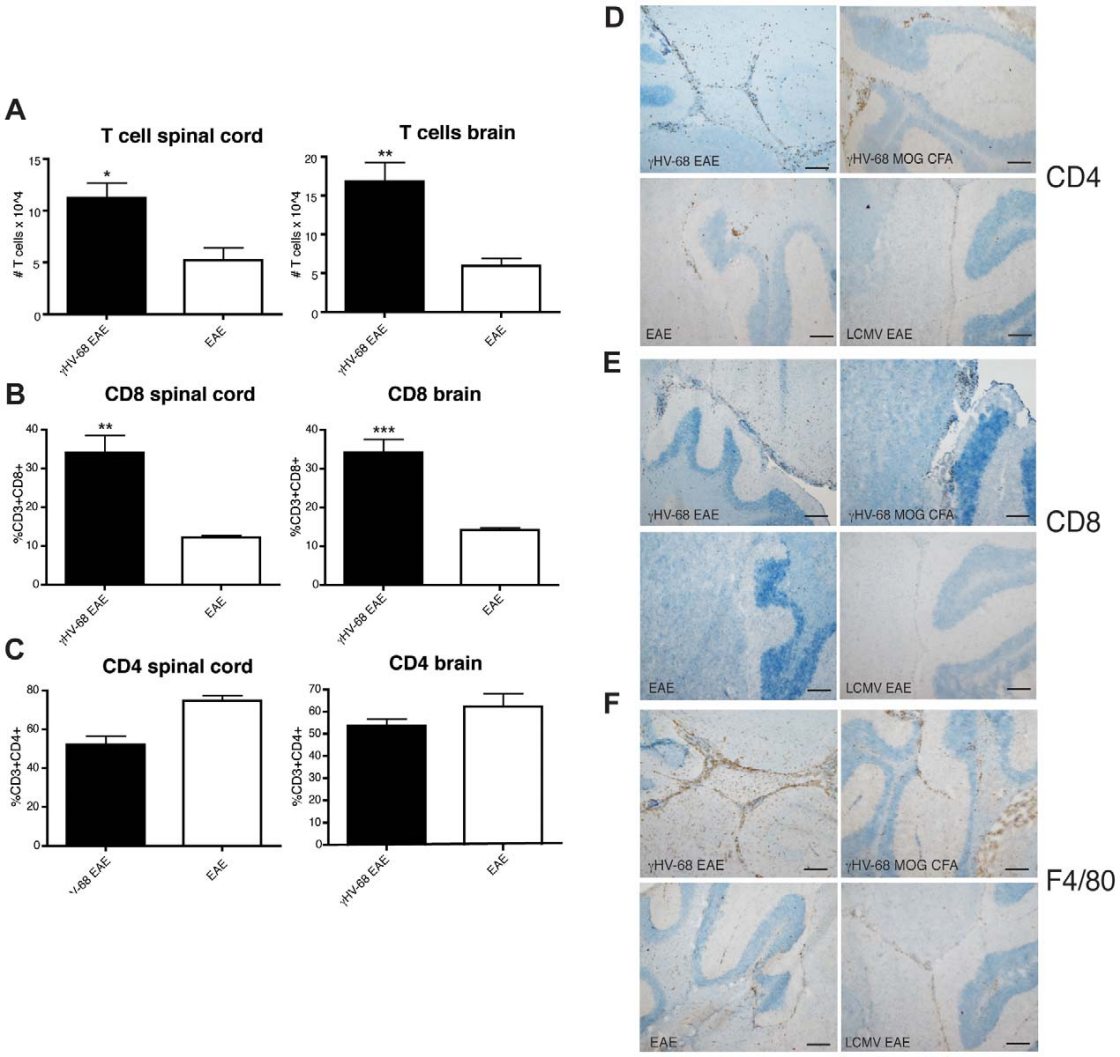
γHV-68 EAE mice show increased amount of CD8 T cells infiltrations in the CNS and inflammation inside the brain parenchyma. Mice were infected with γHV-68 (black bars) or MEM only (open bars). Five weeks p.i. EAE was induced. (**A-B-C**) At day 14–16 post EAE induction (mean clinical score of 3 for γHV-68 EAE mice, EAE mice were harvested at the same time), mice were perfused, brains (right panels) and spinal cords (left panels) were harvested and processed to isolate immune infiltrates. (**A**) Total number of infiltrating T cells (CD45+ CD11b−). (**B**) Percentage of infiltrating CD3+ CD8+ lymphocytes and (**C**) CD3+ CD4+ lymphocytes. Three separate experiments with 8–6 mice/group; data were analyzed with t-test: *** p<0.001; ** p<0.01, * p<0.05. (**D-E-F**) Mice were infected with γHV-68 (upper panels left and right: γHV-68 EAE and γHV-68 MOG CFA); or LCMV (bottom right panel: LCMV EAE) or MEM only (bottom left panel, EAE). Five weeks p.i. EAE was induced with or without (MOG CFA) pertussis toxin. At day 28 post EAE induction (similar results obtained at day 15 post EAE induction), mice were perfused and brains were embedded in OCT, snap frozen, cut and stained with antibodies specific for CD4 (**D**), CD8 (**E**) and F4/80 (**F**). The pictures are representative of three separate experiments (n = 16/group). Scale bar: 100 µm.

Since in addition to severe ascending paralysis, γHV-68 EAE mice also displayed unusual symptoms such as loss of balance, ataxia and hunched posture; we investigated both spinal cord and brain histopathology. During the course of EAE, mice develop ascending paralysis due to spinal cord inflammation. Immune infiltrations in the brain cortex are atypical and, if present, are restricted to the meninges. To assess the composition and to quantify infiltrating immune cells in γHV-68 EAE mice and in EAE mice, CNS infiltrates were isolated and stained at day 14–16 post EAE induction (mean clinical score of 3 for γHV-68 EAE mice, EAE mice were harvested at the same time). γHV-68 EAE mice presented with an increased number of T cells in the CNS, both in the brains and in the spinal cords when compared to EAE mice (Figure 3A). CD4 T cells are the primary T cells type infiltrating the CNS during EAE (Figure 3C). Surprisingly, increased percentages of CD8 T cells were detected in both the spinal cords and the brains of γHV-68 EAE mice (Figure 3B).

**Figure 4:**
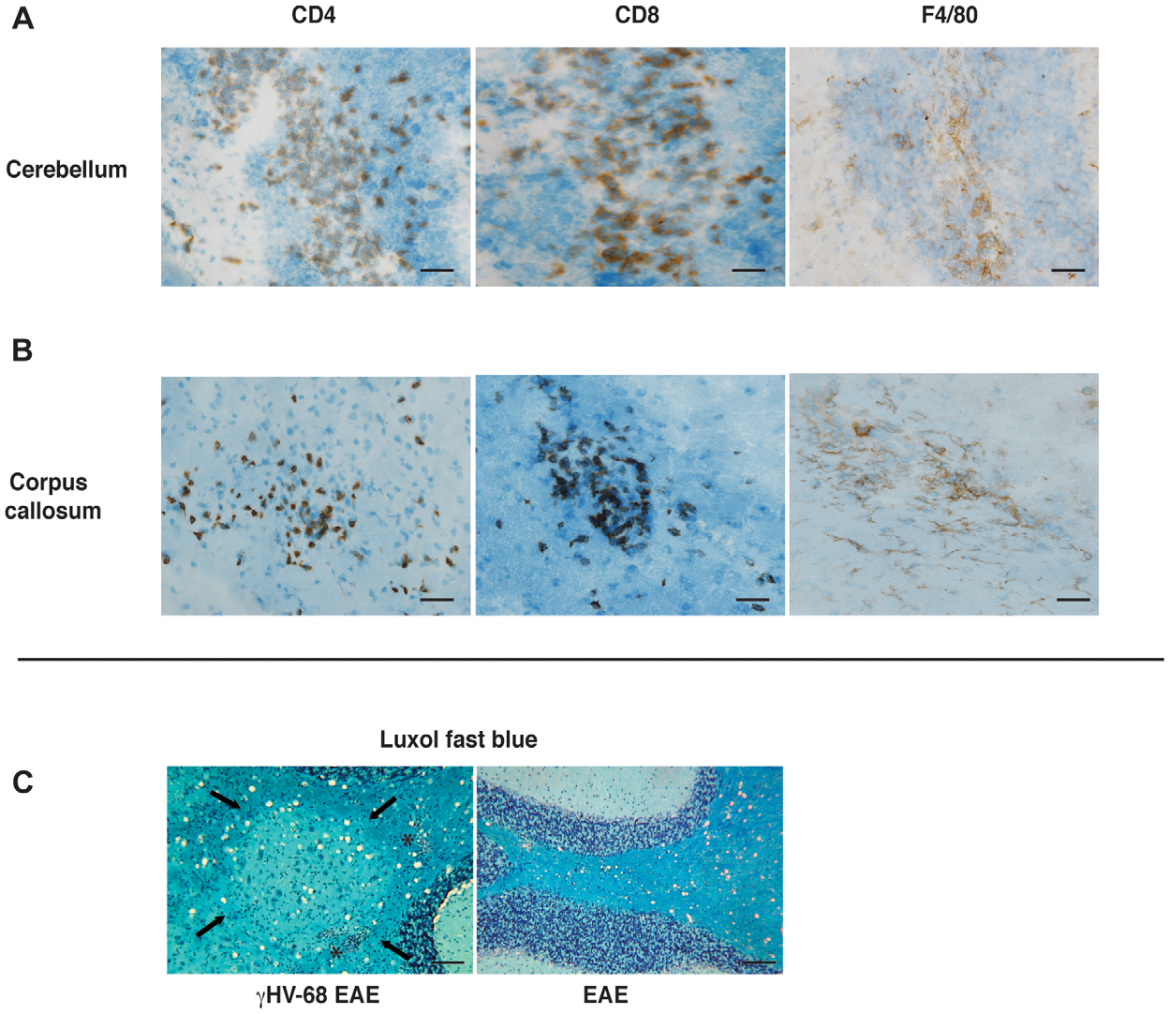
γHV-68 EAE mice show MS-like lesions in the white matter of the cerebellum and in the corpus callosum. Mice were infected with γHV-68 or MEM only. Five weeks p.i. EAE was induced. At day 28 post EAE induction mice were perfused and brains were embedded in OCT, snap frozen, cut and stained with antibodies specific for CD4, CD8 and F4/80; or were formalin fixed and embedded in paraffin and stained with luxol fast blue. (**A–B**) The images displayed for CD4-CD8-F4/80 staining are consecutive sections cut from the same cerebral hemisphere of the same mouse (all images from γHV-68 EAE mice). Scale bar: 50 µm. (**C**) The arrows show an area of demyelination in the cerebellum of a γHV-68 EAE mouse and the asterisks highlight immune cell infiltrations. The right panel shows a normal cerebellum from a naïve EAE mouse. Scale bar: 100 µm. All pictures are representative of two separate experiments (n = 16/group).

To confirm the FACS data and demonstrate that T cells in the brain were infiltrating into the parenchyma and were not confined to the meninges, immunohistochemistry on brain sections was performed both at day 15 and day 28 post EAE induction. Figure 3 shows data from day 28, equivalent results were obtained at day 15 post EAE. γHV-68 EAE mice (upper left panels) showed heightened CD4 (Figure 3D) and CD8 (Figure 3E) T cell infiltrations inside the brain parenchyma. Brain sections were also stained for F4/80 that is expressed both on infiltrating macrophages and on activated microglia, indicating areas of inflammation in the CNS (Figure 3F). γHV-68 EAE mice showed heightened staining in multiple areas of the brain. Conversely, the brains from EAE mice (bottom left panels) showed fewer infiltrating CD4 T cells, mostly lining the blood vessels and the meninges, no CD8 infiltrations and weaker F4/80 staining when compared to brains from γHV-68 EAE mice. Additionally, EAE was induced in γHV-68 mice without administering pertussis toxin (γHV-68 MOG CFA). Pertussis toxin has been shown to be important both as an adjuvant to prime the MOG specific response and to permeabilize the blood-brain barrier [22]. γHV-68 MOG CFA mice develop milder paralysis than γHV-68 EAE mice but still showed T cells infiltrations into the brain parenchyma and inflammation (Figure 3D–F, upper right panels). Further, LCMV EAE mice did not display any observable lymphocyte infiltrations in the brain and had an EAE course equal to that observed in uninfected mice (Figure 3D–F, bottom right panels). Spinal cords were analyzed at the same time points as brains. Hematoxylin and eosin staining showed increased amounts of immune cells infiltrating in the spinal cords of γHV-68 EAE mice, thus confirming the FACS data (Figure S1). Finally and most importantly, the development of lesions in the brain parenchyma similar to MS were observed in γHV-68 EAE mice with localization of infiltrating CD4/CD8 T cells and increased inflammation (F4/80 staining) on consecutive sections (Figure 4). Similar to MS, multiple pronounced yet small lesions of mononuclear cells with areas of myelin loss were observed within the white matter of both the cerebellum (Figure 4) and corpus callosum. This demonstrates that γHV-68 latent infection leads to strong T cell activation post EAE induction with infiltration inside the white matter of the brain leading to myelin damage.

### CD8 T cells infiltrating in the CNS of γHV-68 EAE mice express granzyme B and are specific for viral proteins

**Figure 5:**
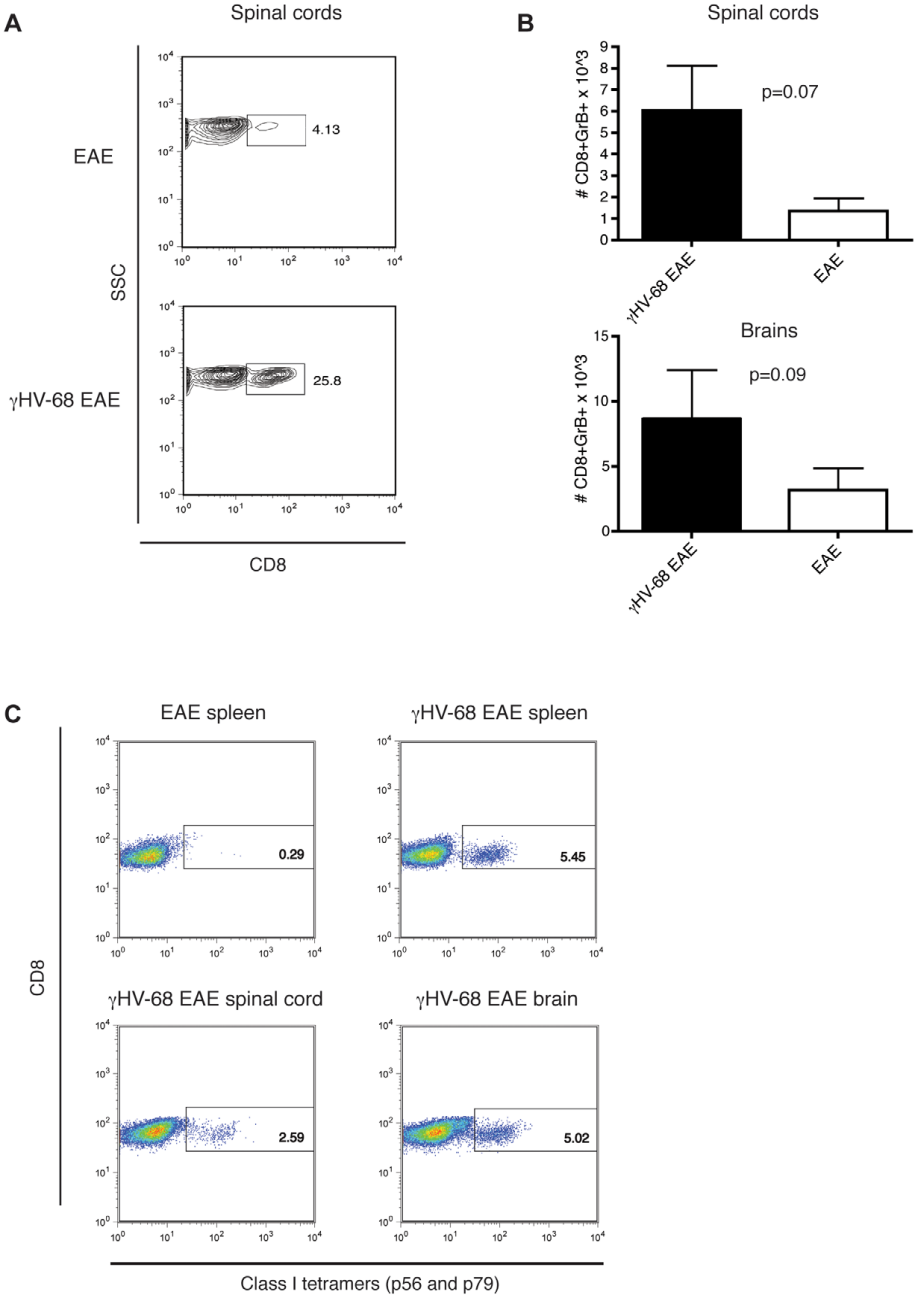
CD8 T cells infiltrating in the CNS of γHV-68 EAE mice express granzyme B and are specific for viral proteins. Mice were infected with γHV-68 or MEM only. Five weeks p.i. EAE was induced. At day 14–16 post EAE induction (mean clinical score of 3 for γHV-68 EAE mice, EAE mice were harvested at the same time), mice were perfused, brains and spinal cords were harvested and processed to isolate immune infiltrates. (**A**) Representative FACS plots showing the percentages of CD8 T cells infiltrating in the spinal cords (similar results were obtained in the brains) and histogram (**B**) showing the number of CD8+ granzyme B+ T cells infiltrating in the spinal cords and in the brains (immune cells were not restimulated before granzyme B staining). Two separate experiments with 6 mice/group, data analyzed with t-test. (**C**) Representative FACS plots showing percentages of CD8+ T cells with TCR specific for γHV-68 epitopes (p56 and p79) in the spleen of EAE mice (negative control); in the spleen of γHV-68 EAE mice (positive control; 3.3±1.6%), in the spinal cord (3.4±0.5%) and in the brain (4.9±0.5%) of γHV-68 EAE mice. Two separate experiments with 6 mice/group, errors are s.d.

CD8 infiltrations, usually not present in the CNS of EAE mice, were detected in the brain and spinal cords of γHV-68 EAE mice in significantly higher percentages (Figure 3 and Figure 5A). CD8 T cells were further characterized for granzyme B production that has the potential to damage oligodendrocytes [23]. γHV-68 EAE mice presented with higher levels of CD8+ Granzyme B+ T cells in both the brain and the spinal cords (Figure 5B).

As it was possible that γHV-68 specific memory CD8 T cells could become bystander activated upon EAE induction and driven into the site of inflammation in the CNS, the specificity of the CD8 in γHV-68 EAE mice was investigated. A percentage of CD8 T cells infiltrating in both the brains (4.9±0.5%) and the spinal cords (3.4±0.5%) of γHV-68 EAE mice (Figure 5C) were specific for the two predominant viral epitopes (p56 and p79). The first epitope is from ORF 6 (p56) that encodes a single stranded DNA binding protein. The second epitope is from ORF 61 (p79) that encodes the large ribonucleotide reductase subunit [24]. Both are expressed during γHV-68 acute infection. The percentages of p56 and p79 specific CD8 T cells detected in the CNS post EAE (49 days postinfection) were equivalent to their respective percentages in spleens (4.3±3.2%) of γHV-68 infected mice at day 40 post primary infection (Figure 5C, upper right panel); consistent with previously published results [24]. These results demonstrate that infiltrating CD8 T cells expressing granzyme B in γHV-68 EAE mice provide a considerable potential for CNS pathology. Moreover, as the size of the population of the virus specific CD8 T cells found in the CNS post EAE was similar to that observed in other organs during latency and typical for latently infected mice 40 days post acute infection, their migration into the CNS is likely due to ordinary activation typically observed following a non-specific environmental insult.

### T cells infiltrating the CNS of γHV-68 EAE mice produce higher amounts of IFN-γ and T-bet along with IL-17 downregulation

**Figure 6:**
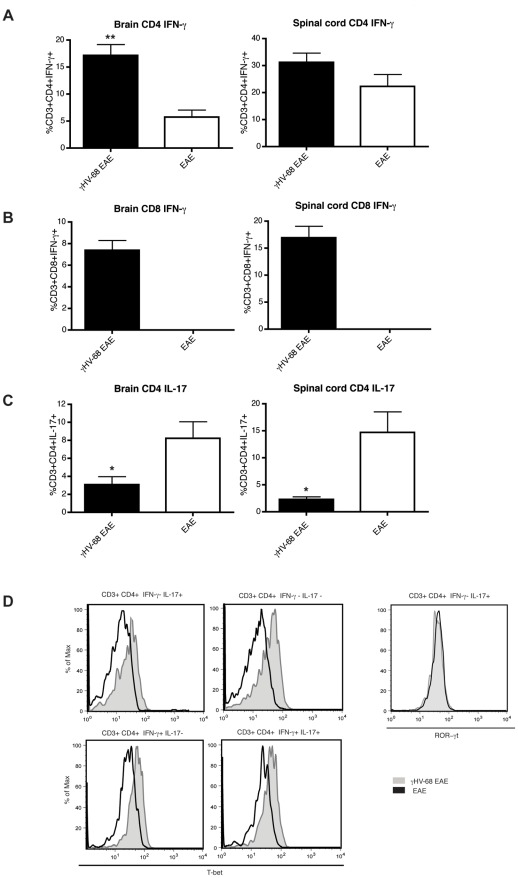
γHV-68 EAE mice show increased T cell expression of IFN-γ and T-bet, accompanied by IL-17 suppression. Mice were infected with γHV-68 (black bars, shaded histograms) or MEM only (open bars, open histograms). Five weeks p.i. EAE was induced. At day 14–16 post EAE induction (mean clinical score of 3 for γHV-68 EAE mice, EAE mice were harvested at the same time), mice were perfused, brains (left panels) and spinal cords (right panels) were harvested and processed to isolate immune infiltrates that were restimulated ex-vivo with PMA and ionomycin before performing FACS intra cellular staining. (**A**) Percentages of infiltrating CD3+ CD4+ IFN-γ+ lymphocytes. (**B**) Percentage of infiltrating CD3+ CD8+ IFN-γ lymphocytes (EAE CD8 infiltrations were not enough to perform FACS). (**C**) CD3+ CD4+ IL-17+ lymphocytes. Three separate experiments with 8-6 mice/group. Data were analyzed with t-test: ** p<0.01, * p<0.05. (**D**) T-bet and ROR-γt levels in CD3+ CD4+ IFN-γ± IL-17± lymphocytes in the spinal cords (similar results obtained from the brains) of γHV-68 EAE mice (grey shaded histograms) or EAE mice (open histograms). Representative histograms of two separate experiments with 6–8 mice/group.

To determine the cytokines produced by the T helper response after EAE immunization, T cells were isolated from the CNS and restimulated ex-vivo (Figure 6). The T cell response primed upon EAE induction in naïve mice is a mixed Th1-Th17 CD4 T cell response with production of both IFN-γ and IL-17 primarily in spinal cords [25]. In contrast, in γHV-68 EAE mice, CD4 T cells produced significantly increased amounts of IFN-γ (Figure 6A), particularly within the brain parenchyma, along with suppressed levels of IL-17 (Figure 6C). Moreover increased percentages of these CNS CD8 T cells from γHV-68 EAE mice produced IFN-γ (Figure 6B). As expected, the amount of CD8 T cells infiltrating the CNS of naïve EAE mice was too low to perform the assay. When restimulated with the MOG specific peptide, similar results were observed with T cells from the CNS (Figure S2). These results indicate that, upon EAE induction in γHV-68 mice, the T helper response was skewed towards a Th1 phenotype, whereas the Th17 response was suppressed.

Additionally, infiltrating T cells were stained for T-bet and ROR-γt (Figure 6D). In γHV-68 EAE mice, T-bet was significantly upregulated in all the CNS infiltrating T cells compared to EAE mice. Strikingly, in γHV-68 EAE mice, T cells not producing IFN-γ (Figure 6D, upper panels) still showed T-bet upregulation. Conversely, ROR-γt was upregulated only in IL-17 secreting T cells and the levels were comparable between EAE and γHV-68 EAE mice (Figure 6D). Finally, γHV-68 T cells, in the periphery, exhibited an effector memory phenotype with increased expression of CD44 and downregulation of CD62L, when compared to T cells from naïve mice either before or after EAE induction (data not shown). This demonstrates that mice latently infected with γHV-68 skew a more potent Th1 response upon immunization with suppression of the classical EAE Th17 response.

### γHV-68 EAE mice have increased levels of pro-inflammatory cytokines in the serum and a decreased anti-MOG IgG1/IgG2a ratio

**Figure 7:**
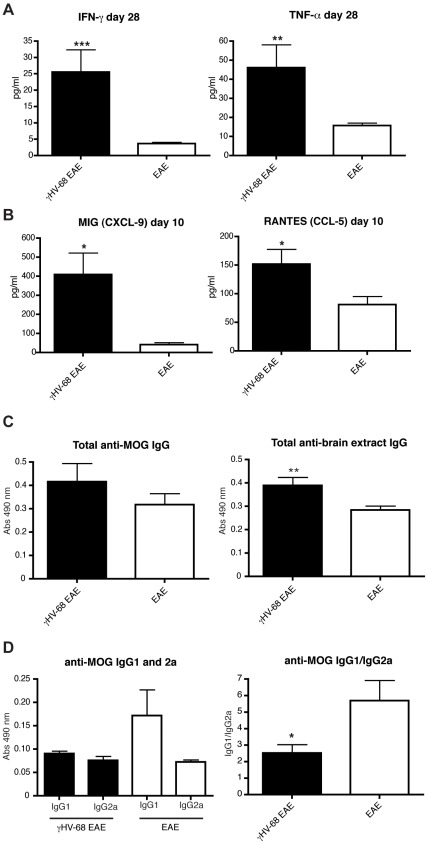
γHV-68 EAE mice show increased levels of pro-inflammatory cytokines and chemokines and a decreased anti-MOG IgG1/IgG2a ratio in the serum. Mice were infected i.p. with γHV-68 (black bars) or MEM only (open bars). Five weeks p.i., EAE was induced. At day 10, 15 and 28 post EAE induction blood was harvested through a cardiac puncture and the levels of different (**A**) cytokines (day 28 shown here) and (**B**) chemokines (day 10 shown here) were evaluated using BD Cytometric Bead Array kits. Data from day 28 are shown for both IFN-γ and TNF-α; similar results were obtained at day 10 and 15 post EAE induction. Three-two separate experiments for each time point with 3–6 mice/group. Data were analyzed with t-test: *** p<0.001; ** p<0.01, * p<0.05. (**C–D**) Serum was harvested at day 28 post EAE and the levels of (**C**) total anti-MOG IgG (left panel), total anti-brain extract IgG (right panel) and (**D**) anti-MOG IgG1 and IgG2a were quantified through ELISA. Two separate experiments with 8 mice/group. Data were analyzed with t-test: ** p<0.01; and Mann-Whitney U test: * p<0.05.

Since γHV-68 EAE mice showed a strong Th1 response, the level of Th1 cytokines in the serum was measured. Sera was harvested at different time points post EAE induction and the following cytokines and chemokines were analyzed: IFN-γ, IL-12p70, TNF-α, IL-6, IL-10, IL-17A, GM-CSF, G-CSF, MCP-1 (CCL2), MIP-1α (CCL-3), MIP-1β (CCL-4), MIG (CXCL-9) and RANTES (CCL-5). IFN-γ and TNF-α were significantly increased in the serum of γHV-68 EAE mice, when compared to EAE mice, at day 10, 15 and 28 post EAE induction (Figure 7A shows results for day 28, similar results were obtained at day 10 and 15 post EAE, as shown in Figure S3). On the other hand, the chemokines RANTES (CCL-5) and MIG (CXCL-9) were statistically significant different only at day 10 post EAE induction, when mice started to develop symptoms (Figure 7B). All the remaining cytokines tested and listed above were not differentially expressed between the sera of the two mouse groups. Levels of cytokines and chemokines were also analyzed in the supernatants obtained from brain and spinal cords homogenates (see Materials and Methods for CNS supernatant preparation). At day 14–16 post EAE induction, MCP-1 was detected in the brains and spinal cords of both EAE and γHV-68 EAE mice at similar levels. Increased levels of IFN-γ were detected in the brains and spinal cords of γHV-68 EAE mice when compared to EAE mice (Figure S4). All the remaining cytokines analyzed in the CNS were below the detection limit. Considering the importance of type I interferons in controlling acute γHV-68 infection [26], the levels of IFN-β in the sera were also tested. IFN-β was detected at a similar level in the sera of both γHV-68 EAE mice and uninfected EAE mice (data not shown).

Since CCL-5 and CXCL-9 were found to be upregulated in the sera of γHV-68 EAE mice, the levels of their respective chemokine receptors (CCR5 for CCL-5 and CXCR3 for CXCL-9) were analyzed at day 15 post EAE induction in the spleen. No difference was detected in the expression of CCR5 in both CD4 and CD8 T cells. On the other hand, greater numbers of CD4 and CD8 T cells from γHV-68 EAE mice expressed CXCR3, compared to cells from uninfected EAE mice (Figure S5).

These results show that the Th1 response triggered by EAE induction in γHV-68 EAE mice is sustained even at later time points post immunization and high levels of IFN-γ and TNF-α are likely responsible of perpetuating this Th1 response. Moreover, as RANTES and MIG control leukocyte adhesion and migration into the target tissue [27], their upregulation at day 10 post EAE, coincident with the upregulation of CXCR3 on T cells, is likely responsible for the increased T cell infiltration in γHV-68 EAE mice.

Since γHV-68 infects B cells and B cells infiltrating the CNS were not detected (results confirmed by both FACS analysis of CNS infiltrates and immunohistochemistry at day 15 and 28 post EAE using anti-CD19 antibodies), the hypothesis that the viral infection could precipitate EAE by increasing the production of anti-MOG autoantibodies was investigated. Sera harvested at day 28 was tested for the presence of both MOG-specific and brain-extract specific IgGs (Figure 7c). There was no difference in the amount of anti-MOG IgG but there was an increase in the amount of anti-brain extract antibody in γHV-68 EAE mice. This indicates that MOG autoantibodies are not likely playing a role in the increased clinical score of γHV-68 EAE mice and the increased presence of brain-extract specific antibodies are likely due to epitope spreading to CNS proteins due to increased CNS inflammation in γHV-68 EAE mice. Since anti-MOG IgGs were not different between infected and uninfected mice, differences in IgG isotype were tested. γHV-68 EAE mice showed a decreased anti-MOG IgG1/IgG2a ratio (Figure 7D). This is consistent with the high concentrations of IFN-γ detected in the serum that are likely inhibiting isotype switching towards IgG1 [28], thus confirming the presence of a strong Th1 response in γHV-68 EAE mice.

### CD11b+ CD11c+ cells from γHV-68 EAE mice are able to prime an enhanced Th1 response both in vitro and in vivo

**Figure 8:**
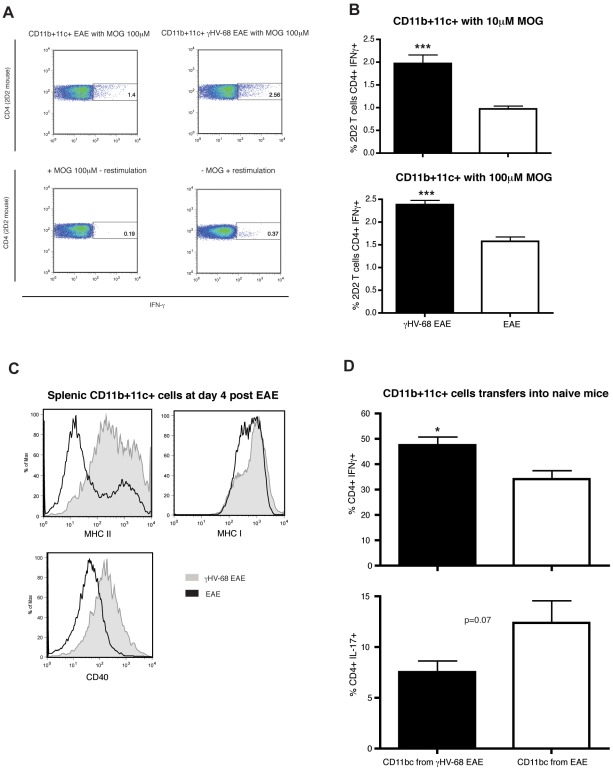
CD11b+ CD11c+ cells from γHV-68 EAE mice are able to prime an enhanced Th1 response both in vitro and in vivo. Mice were infected with γHV-68 or MEM only. Five weeks p.i., EAE was induced. At day 4 post EAE induction, spleens and lymph nodes were harvested and CD11b+CD11c+ cells were isolated. CD4 T cells from 2D2 mice were isolated at the same time. CD11b+CD11c+ were incubated with 2D2 CD4 T cells and different concentrations of MOG peptide for 72 hours. T cells were restimulated and stained to assess the production of IFN-γ (**A**) Representative FACS plot showing IFN-γ production in 2D2 CD4 T cells after incubation with CD11b+CD11c+ cells isolated from a γHV-68 EAE mouse (upper right panel) or a naïve EAE mouse (upper left panel) and MOG peptide (100 µM). Lowers panels show the negative controls with/without MOG and with/without restimulation. (**B**) Histograms showing the percentages of 2D2 CD4 T cells producing IFN-γ after incubation with CD11b+CD11c+ cells from a γHV-68 EAE mouse (black bars) or a naïve EAE mouse (open bars) and 10 µM (left panel) or 100 µM (right panel) MOG peptide. Three separate experiments with triplicate wells for each group. Data were analyzed with t-test: *** p<0.001. (**C**) Levels of MHC II, MHC I and CD40 expressed on CD11b+CD11c+ isolated from the spleens of γHV-68 EAE mice (grey shaded histograms) or a naïve EAE mice (open histograms) at day 4 post EAE induction. (**D**) CD11b+CD11c+ isolated from both γHV-68 EAE and naïve EAE mice at day 4 post EAE were transferred into naïve mice. Twenty-four hours later EAE was induced in both groups. At day 14–16 post EAE induction (mean clinical score between 2 and 3 for both groups), brains and spinal cords were harvested and the production of IFN-γ and IL-17 by CD4 T cells in the CNS of mice that received CD11b+CD11c+ cells from γHV-68 EAE mice (black bars) or from naive EAE mice (open bars) was assessed after in-vitro restimulation with PMA and ionomycin. Results obtained from the spinal cords are shown, a similar trend was observed in the brain. Three separate experiments with 5 mice/group. Data were analyzed with t-test: * p<0.05.

Collectively our results indicate that γHV-68 EAE mice are polarizing a distinct T helper response post MOG immunization in comparison with typical EAE. We hypothesized that a subset of antigen-presenting cells, as a result of a direct infection or under the influence of other signals received by other immune cells during the infection, were able to skew a Th1 phenotype upon MOG presentation. The levels of different cytokines produced by B cells, CD11b+ and CD11c+ cells were assessed during the antigen presentation phase of EAE (day 4). In γHV-68 EAE mice, CD11b+ CD11c+ cells produced IFN-γ at day 4 post EAE (7–8%); conversely, CD11b+CD11c+ cells from uninfected EAE mice produced less IFN-γ (3–4%). To test the ability of these antigen-presenting cells to prime a Th1 phenotype during MOG presentation, T cells from 2D2 T cell receptor (TCR) transgenic mice, that express a MOG- specific TCR, were isolated and incubated with MOG peptide and CD11b+ CD11c+ cells isolated either from an uninfected EAE mouse or a γHV-68 EAE mouse at day 4 post EAE induction. γHV-68 EAE CD11b+ CD11c+ cells induced an increased production of IFN-γ in these MOG specific transgenic T cells (Figure 8A–B), while IL-17 was not detectable. Further, a nested PCR on CD11b+ CD11c+ cells isolated from γHV-68 mice did not find any γHV-68 DNA, indicating that the ability of these antigen-presenting cells to prime a Th1 response was not dependent on direct γHV-68 infection or replication. Additionally, the expression levels of co-stimulatory molecules and MHC class I- II on CD11b+ CD11c+ cells were analyzed at day 4 post EAE induction. Splenic CD11b+CD11c+ cells isolated from γHV-68 EAE mice expressed higher levels of CD40 and MHC I -II when compared to EAE mice (Figure 8C), whereas the levels of CD80 and CD86 were similar between the two groups. Finally, to assess if these CD11b+CD11c+ cells were able to prime a stronger Th1 response also in vivo, CD11b+CD11c+ cells isolated from either γHV-68 EAE mice or EAE mice were adoptively transferred into naïve mice and 24 hours later EAE was induced. Mice transferred with γHV-68 EAE CD11b+CD11c+ cells presented with increased percentages of infiltrating CD4+ IFN-γ+ T cells into the CNS and decreased percentages of CD4+ IL-17+ T cells (Figure 8D shows data obtained from the spinal cords, a similar trend was observed in the brains). These results indicate that a subset of CD11b+ CD11c+ antigen-presenting cells found in γHV-68 EAE mice, without being directly infected by the virus, induced the production of increased levels of IFN-γ in T cells both in vivo and in vitro. This cell subset is likely responsible for the polarization of the potent Th1 response that is observed in γHV-68 EAE mice.

## Discussion

The study of the “viral etiology of MS" has been challenging because of the absence of a mouse model that could be exploited to dissect the relationship between candidate MS viruses and development of CNS autoimmunity. EBV has been strongly linked to MS development even if the mechanisms by which EBV triggers autoimmunity are not understood [29]. We demonstrated that a latent infection with a gamma herpesvirus changes the polarization of the myelin specific T cell response after EAE induction leading to a potent Th1 response with more severe paralysis, atypical neurological symptoms, different composition and localization of the CNS infiltrates and myelin lesions in the white matter reminiscent of human MS.

Brain inflammation accompanied by T cell infiltrations within the brain parenchyma is rarely detected in EAE, although it is a critical characteristic of human MS [1]. Further, during EAE, CD4 T cells are the predominant T cell type to invade the CNS, whereas in humans, CD8 T cells are equally present in the MS plaques [1], [17]. In γHV-68 EAE mice, CD8+ IFN-γ+ granzyme+ cells are infiltrating the brain parenchyma and a minority of these is specific for γHV-68 proteins. Thus, activated CD8 T cells that are not specific for CNS epitopes are able to enter the brain parenchyma and likely participate in sustaining a pro-inflammatory loop that recruits additional immune cells. These findings are similar to those recently shown by Matullo et al. in which a polymicrobial challenge leads to the recruitment of LCMV specific T cells in the CNS despite the absence of LCMV infection in the CNS [30]. We suggest that our model could potentially be used to study the behavior of CD8 T cells in CNS autoimmunity and elucidate the signals that guide CD8 infiltrations inside the brain during demyelinating diseases.

It has long been described that MS patients have a potent CNS Th1 response [31]–[32]. The potent Th1 response observed in γHV-68 EAE mice not only includes higher amounts of IFN-γ but also an upregulation of T-bet in all T cells, including those not actively producing any cytokines. Interestingly T cells from MS patients exhibit preferentially a Th1 phenotype [31]–[32], T-bet levels are predictive of IFN-β therapy efficacy in MS patients [33] and T-bet is upregulated in patients with active disease and is downregulated during remission [34].

Our data demonstrate that, upon MOG peptide presentation, CD11b+CD11c+ from γHV-68 EAE mice are able to induce, both ex vivo and in vivo, an increased production of IFN- γ in T cells with a suppression of the Th17 response. Intriguingly, CD11b+CD11c+ from γHV-68 EAE mice have increased expression of MHC class I and II as well as CD40. It has been shown that the strength of signal upon antigen presentation dictates the fate of the T helper response [35]. A weaker signal preferentially primes a Th17 response. Thus, increased expression of MHC II and CD40 on the surface of CD11b+CD11c+ cells likely provides a strong activation signal and is likely responsible for the suppression of the Th17 response and the skewing towards the observed Th1 phenotype found in γHV-68 EAE mice. CD11b is found mainly on the surface of monocytes/macrophages, but it can also be co-expressed, along with CD11c, on the surface of dendritic cells and NK cells [36]–[37]. Moreover, IFN-γ is mainly produced by T cells and NK cells but also macrophages [38] and dendritic cells [39] have been shown to produce IFN-γ in response to both endogenous and exogenous stimuli. Interestingly, γHV-68 activates macrophages and this phenotype protects mice from lethal infections by another intracellular pathogen, Listeria [40]. This mechanism is independent from T cells but dependent on IFN-γ, showing that a latent infection with γHV-68 was conferring a broad innate cross protection that did not require IFN-γ produced by memory T cells. In our model, it is possible that macrophages and/or dendritic cells with an activated phenotype that are secreting more pro-inflammatory Th1 skewing cytokines are responsible for the priming of the potent Th1 response in γHV-68 EAE mice.

Lastly and significantly, the ability to discover EBV in the brains of MS patients has been controversial [11]–[14]. In the γHV-68 EAE mouse model, γHV-68 is no longer detectable in the CNS tissue (by PCR) during disease and yet, it is indirectly influencing the autoimmune response and immune cell polarization. It is easy to imagine that EBV may well be acting similarly as the first trigger of the second hit hypothesis. It is intriguing that mice latently infected with the murine homologue of EBV are developing an EAE that is more reminiscent of human MS. From this model, experiments can be designed to ask how γHV-68 is acting to allow T cells to preferentially enter the brain during MS, specifically addressing the signals that are required and where they arise.

In conclusion, we propose that EBV latent infection in patients is influencing the development of disease following a second hit. In this case, EAE induction results in a stronger disease as the result of polarization of the adaptive T cell response. In MS patients, a second trigger also leads to the skewing of the immune system towards a Th1 biased phenotype and an increased activation status resulting in MS lesions. As such, a patient's history of infection and, specifically, EBV latent infection are as important as genetics in determining an individual's susceptibility to autoimmune diseases.

## Materials and Methods

### Ethics statement

All animal work was performed under strict accordance with the recommendations of the Canadian Council for Animal Care. The protocol was approved by the Animal Care Committee (ACC) of the University of British Columbia (certificate numbers: A08-0415 and A08-0622).

### Infections and EAE induction

C57Bl/6 mice and 2D2 mice were purchased from the Jackson Laboratory and were bred and maintained in our rodent facility at the University of British Columbia. Mice were infected intraperitoneally (i.p.) between 7–10 weeks of age with 10^4^ pfu of γHV-68 WUMS strain (purchased from ATCC, propagated on BHK cells); or 10^4^ pfu of LCMV Armstrong strain 53b (originally acquired from Dr. M.B. Oldstone, propagated on BHK cells); or 2,500 pfu of MCMV (from ATCC, generous gift of Dr. S.M. Vidal); or 200 µl of MEM as a control. Cidofovir (Vistide; Gilead Sciences) was diluted in PBS and filter sterilized. It was administered subcutaneously in the scruff of the neck at a dose of 25 mg/kg or 15 mg/kg as previous reports [21], [41]. Mice were given a two days loading dose four weeks post γHV-68 infection and 10 days before EAE induction. After, mice were injected every 3^rd^ day until day 15 post EAE induction when they were euthanized. EAE was induced 35–40 days post infection by injecting subcutaneously each mouse with 100 µl of emulsified complete Freund's adjuvant (DIFCO) with 200 µg of MOG 35–55 (GenWay biotech, purity >95%) and 400 µg of dessicated Mycobacterium tuberculosis H37ra (DIFCO). Mice also received two i.p. injections with 200 ng of pertussis toxin (List Biologicals) at time of immunization and 48 hours later. Mice were scored on a scale of 0 to 5: 0, no clinical signs; 0.5, partially limp tail; 1, paralyzed tail; 2 loss of coordinated movements; 2.5; one hind limb paralyzed; 3, both hinds limbs paralyzed; 3.5, hind limbs paralyzed, weakness in the forelimbs; 4, forelimbs paralyzed; 5, moribund or dead.

### Limiting dilution assay

At indicated time points post EAE, mice were euthanized and spleen harvested. A single cell suspension was generated after RBC lysis. A limiting dilution assay was performed, as previously described [42], to analyze the amount of ex-vivo reactivation (using live splenocytes) and the amount of pre-formed virus (using lysed splenocytes through one cycle of freeze-thaw). Splenocytes were plated on a 96 well plate on a monolayer of mouse embryonic fibroblasts (MEF). Twelve 2-fold serial dilutions were prepared starting at 10^5^ cells/well. Twelve wells were plated for each dilution. Plates were incubated at 37°C for 15–20 days and then scored for cytopathic effect.

### Nested PCR

DNA was extracted from brains, spinal cords and spleens of perfused mice at indicated time points before and post EAE induction using QIAamp DNA mini kit (QIAGEN) following manufacture's instruction. A nested PCR to detect γHV-68 was performed as previously described [43]. Briefly, 2 µl of DNA were added to a PCR mix composed of 0.2 mM dNTPs, 0.4 µM primers and 2.5 U of Taq polymerase. The PCR cycles were the following: 95°C for 2 min, followed by 20 cycles at 95°C for 1 min, 63°C for 1 min and 72°C for 1 min followed by 7 minutes at 72°C. The primer used were 5′ CCA TCT AGC GGT GCA ACA TTT TCA TTA C 3′ (forward) and 5′ TTT ACT GGG TCA TCC TCT TGT TTG GG 3′ (reverse). Then, 2.5 µl from the previous PCR reaction were used for the second PCR reaction using the following internal primers with the same cycles: 5′ CGA ACA ACA ATC CCA CTA CAA TTA TGC G 3′(forward) and 5′ GTA TCT GAT GTG TCA GCA GGA GCG TC 3′ (reverse). The samples were run on a 2% agarose gels (Invitrogen) and the 462 bp expected band was visualized using SYBR safe (Invitrogen).

### Immune cells isolation, staining and flow cytometry

At day 14–16 post EAE induction (mean clinical score of 3 for γHV-68 EAE mice), mice were perfused with 30cc of PBS and spinal cords, brains, spleens, inguinal and cervical lymph nodes were isolated at indicated time points post EAE induction. A single cell suspension was generated from all the organs. Immune cells were further isolated from the CNS using a 30% Percoll gradient. For intracellular staining, CNS mononuclear cells, splenocytes and lymph nodes cells were stimulated for 4 hours in IMDM (Gibco) containing 10% FBS (Gibco), GolgiPlug (BD Biosciences), 10 ng/ml PMA and 500 ng/ml ionomycin; or for 24 hours with MOG peptide (100 µM) with addition of GolgiPlug during the last 5 hours of incubation. Cells were then incubated with Fc block (BD Biosciences) on ice for 15 minutes. Antibodies for the cell surface markers were added to the cells in PBS with 2% FBS for 30 min on ice. After wash, cells were resuspended in Fix/Perm buffer (eBiosciences) for 30–45 min on ice, washed twice and incubated with Abs for intracellular antigens (cytokines and transcription factors) in Perm buffer (30 min, on ice). Fluorescently conjugated antibodies directed against CD11b (clone M1/70), CD11c (clone N418), CD4 (clone RM4-5), CD8 (clone 53-6.7), CD19 (clone eBio1D3), CD3 (clone eBio500A2), CD45 (clone 30-F11), CD44 (clone IM7), CD62L (clone MEL-14), CD80 (clone 16-10A1), CD86 (clone GL1), CD40 (clone 1C10), MHC I (clone 28-14-8), MHC II (clone M5), IL-6 (clone MP5-20F3), IL-10 (clone JES5-16E3), IL-17 (eBio17B7), IFN-γ (clone XMG1.2), TNF-α (clone MP6-XT22), T-bet (clone eBio4B10), ROR-γt (clone AFKJS-9), granzyme B (clone 16G6) were all purchased from eBiosciences. Anti IL-12 antibody (clone C15.6) was purchased from BD Biosciences. Anti-CCR5 (clone HM-CCR5) and CXCR3 antibodies (clone CXCR3-173) were purchased from Biolegend. Samples were acquired using a FACS LSR II (BD Biosciences) and analyzed using FlowJo software (Tree Star, Inc).

### Tetramer staining

Immune cells were isolated as detailed above. One million cells were incubated with a mixture of two class I tetramers, both conjugated with APC (provided by the Trudeau Institute Molecular Biology Core Facility). Tetramer p56 (D^b^/ORF6_487–495_ AGPHNDMEI) was diluted 1∶300 and tetramer p79 (K^b^/ORF61_524–531_ TSINFVKI) was diluted 1∶400. Cells were incubated for 1 hour at RT and then washed. Surface staining was then performed and cells were fixed in 1% PFA for 20 min on ice before acquisition.

### Histology and immunohistochemistry

Spinal cords harvested from perfused mice were formalin fixed and paraffin embedded. Six-micron thick sections were stained with eosin and hematoxylin or luxol fast blue (all from Sigma) following standard protocols. Brains from perfused mice were frozen in OCT (Fisher Scientific) and ten-micron thick sections were processed for immunohistochemistry. Briefly, sections were fixed in ice cold 95% ethanol for 15 min and washed in PBS several times. This was followed by washes in TBS with 0.1% Tween (TBST) and incubation for 10 min with 3% H_2_O_2_ to block endogenous peroxidase. After washing, blocking buffer was added for 1 h (10% normal goat serum in PBS). Primary antibody was added overnight at 4°C: purified rat anti-mouse CD4, anti-mouse CD8 and anti-mouse F4/80 (all from eBiosciences), diluted 1∶100 in PBS 2% normal goat serum. After washes in TBST, the biotinylated secondary antibody (anti-rat IgG, mouse absorbed, Vector) was added for 1 h, diluted 1∶200 in PBS 2% normal goat serum. After washes in TBST, the Vectastain ABC reagent was used (Vector) following manufacturer's instruction. Then, DAB (Sigma) was added as a substrate and, after incubation for 8 min in the dark and several washes in distilled water, sections were counterstained with Harris hematoxylin for 20 seconds, in lithium carbonate for 30 sec, washed in several changes of distilled water and mount with VectaMount AQ (Vector). Images were acquired at RT using an Olympus BX61 microscope (4×–20× UPLSAPO objective lenses) equipped with an Olympus DP72 digital camera, using the CellSens dimension software.

### Cytokines and chemokines analysis

Serum cytokine and chemokine levels were measured at the indicated timepoints using a mouse inflammation CBA kit (BD Bioscience) for detection of IL-6, IL-10, MCP-1, IFN-γ, TNF-α and IL-12p70 or a cytokine flex set (BD Bioscience) allow for detection of CXCL-9 (MIG), CCL-3 (MIP-1α), CCL-4 (MIP-1β), CCL-5 (RANTES), IL-17A, GM-CSF and G-CSF. Samples were prepared according to manufacturer's instructions and analyzed on a BDFacsArray equipped with FCAP software (BD Biosciences). Serum levels of IFN-β were measured using a VeriKine Mouse interferon beta kit (PBL interferon source) according to manufacturer's instructions. For CNS supernatant analysis, brains and spinal cords were homogenized in 10 mls of FACS buffer to obtain a single cell suspension. The suspension was then centrifuged for 10 min at 1200 rpm. A total of 10 mls of CNS supernatant was obtained for each sample, 1 ml was frozen at −80°C and then 50 µl were analyzed using the mouse inflammation CBA kit as detailed above.

### Auto-antibodies ELISA

Nunc immunoplate were coated with 10 µg/ml of MOG peptide or 10 µg/ml of brain extract (purchased from Santa Cruz Biotechnology) in carbonate buffer and incubated overnight at 4°C. Plates were washed three times with wash buffer (PBS, 0.05% Tween). Blocking buffer (PBS with 1% BSA; 0.05% Tween and 10% FBS) was added in each well for 1 h. After removal of the blocking buffer, day 28 post EAE induction sera samples diluted 1∶20 and 1∶40 in blocking buffer were added for 1.5 h at RT. After washing, anti-mouse IgG-HRP (Sigma), diluted 1∶1000 in blocking buffer, was added for 1 h. After washing, OPD (0.2 mg/ml, Sigma) and UPO (0.2 mg/ml) in citrate buffer were added for 30 min and the reaction was stopped with 25% H_2_SO_4_. Absorbance was read at 490 nm. For IgG1/IgG2a ELISA the following antibodies were used: biotin anti-mouse IgG1 (diluted 1∶10000 in blocking buffer) and biotin anti-mouse IgG2a (diluted 1∶6250 in blocking buffer), both from Jackson Immunoresearch (generous gift of Dr. J. Quandt). In this case, after washing and before adding the substrate, streptavidin-HRP was added (diluted in blocking buffer 1∶1000, Jackson Immunoresearch).

### Direct intracellular staining, mixed assay and cell transfers

Sterile Brefeldin A was purchased from Sigma and 250 µg were injected intravenously in mice at day 4 post EAE induction [44]. Six hours later mice were euthanized and spleen and inguinal lymph nodes were harvested. Cells were prepared and stained to detect IL-6, IL-12, TNF-α, IFN-γ and IL-10 as described above. Intracellular staining was performed without any further ex-vivo restimulation.

For mixed assay, spleens and inguinal lymph nodes were harvested at day 4 post EAE induction. A single cell suspension was prepared and stained with anti-CD11c and anti-CD11b antibodies (see above for details). CD11c+ CD11b+ cells were sorted with a FACSAria cell sorter (BD Biosciences). CD4 T cells from 2D2 mice were isolated from spleens with a CD4 T cells negative selection kit following manufacturer's instructions (STEMCELL technologies). Isolated CD11b+CD11c+ (2×10^4^/well) and 2D2 CD4 T cells (5×10^5^/well) were seeded on a 24 well plate in RPMI, 10% FBS and Pen/Strep (all from GIBCO) with or without 10 or 100 µM MOG peptide. After 72 hours, T cells were restimulated with PMA, ionomycin and GolgiPlug and stained for CD4 and IFN-γ as described above.

For cell transfers, CD11b+CD11c+ cells were sorted as described above. Each mouse received 100,000 cells intra peritoneally. EAE was induced 24 hours post-transfer.

### Statistical analysis

Two-way ANOVA analysis followed by Bonferroni's post test was used to compare EAE scores, Kaplan-Meier analysis was used to compare survival curves and unpaired Student's t-test or Mann-Whitney U-test were used for all the other analyses (GraphPad Prism).

## Supporting Information

Figure S1 **γHV-68 EAE mice show increased amount of immune cells infiltrations in the spinal cords.** Mice were infected with γHV-68 (right panels) or MEM only (left panels). Five weeks p.i. EAE was induced. At day 28 post EAE induction mice were perfused and spinal cords were harvested, fixed in formalin and paraffin embedded (similar results obtained at day 15 post EAE induction). Cross-sections were stained with H&E (upper panels) and luxol fast blue (lower panels). Representative pictures of three separate experiments. Scale bar = 100 µm.
(TIF)

Figure S2 **γHV-68 EAE mice show increased T cell expression of IFN-γ accompanied by IL-17 suppression after MOG restimulation.** Mice were infected with γHV-68 (black bars, shaded histograms) or MEM only (open bars, open histograms). Five weeks p.i. EAE was induced. At day 14–16 post EAE induction (mean clinical score of 3 for γHV-68 EAE mice, EAE mice were harvested at the same time) mice were perfused, brains (left panels) and spinal cords (right panels) were harvested and processed to isolate immune infiltrates that were restimulated for 24 hours with 100 µM MOG peptide before performing FACS intra cellular staining (**A**) Percentages of infiltrating CD3+ CD4+ IFN-γ+ lymphocytes. (**B**) Percentage of infiltrating CD3+ CD8+ IFN-γ lymphocytes (EAE CD8 infiltrations were not enough to perform FACS). (**C**) CD3+ CD4+ IL-17+ lymphocytes. Two experiments with 6 mice/group. Data were analyzed with t-test: * p<0.05.
(TIF)

Figure S3 **γHV-68 EAE mice show increased levels of pro-inflammatory cytokines in the serum.** Mice were infected i.p. with γHV-68 (black bars) or MEM only (open bars). Five weeks p.i., EAE was induced. At day 10 (**A**) and 15 (**B**) post EAE induction blood was harvested through a cardiac puncture and the levels of cytokines were evaluated using BD Cytometric Bead Array kits. Three-two separate experiment for each time point with 3–6 mice/group. Data were analyzed with t-test: *** p<0.001; ** p<0.01, * p<0.05.
(TIF)

Figure S4 **γHV-68 EAE mice show increased levels of IFN-γ in CNS supernatants.** Mice were infected i.p. with γHV-68 (black bars) or MEM only (open bars). Five weeks p.i., EAE was induced. At day 14–16 post EAE induction (mean score of 3 for γHV-68 EAE mice, EAE mice were harvested at the same time), mice were perfused and brains and spinal cords were homogenized and the supernatants were analyzed for the presence of cytokines. Levels of cytokines were evaluated using BD Cytometric Bead Array kits. Two separate experiments with 3–6 mice/group. Data were analyzed with t-test: * p<0.05.
(TIF)

Figure S5 **γHV-68 EAE mice show increased levels of CXCR3 on splenic T cells.** Mice were infected i.p. with γHV-68 (black bars) or MEM only (open bars). Five weeks p.i., EAE was induced. At day 15 post EAE induction, spleens were harvested and the levels of CXCR3 were assessed through FACS analysis. The histograms show the numbers of CD4+CXCR3+ cells (left panel) or CD8+CXCR3+ cells (right panel) One experiment with 5–6 mice/group. Data were analyzed with t-test: *** p<0.001; ** p<0.01.
(TIF)
