# Supplementary figures and images for: Gammaherpesvirus Latency Accentuates EAE Pathogenesis: Relevance to Epstein-Barr Virus and Multiple Sclerosis
Source: PLoS Pathog. 2012 May 17;8(5):e1002715. doi: 10.1371/journal.ppat.1002715 (PMC3355105; doi:10.1371/journal.ppat.1002715)

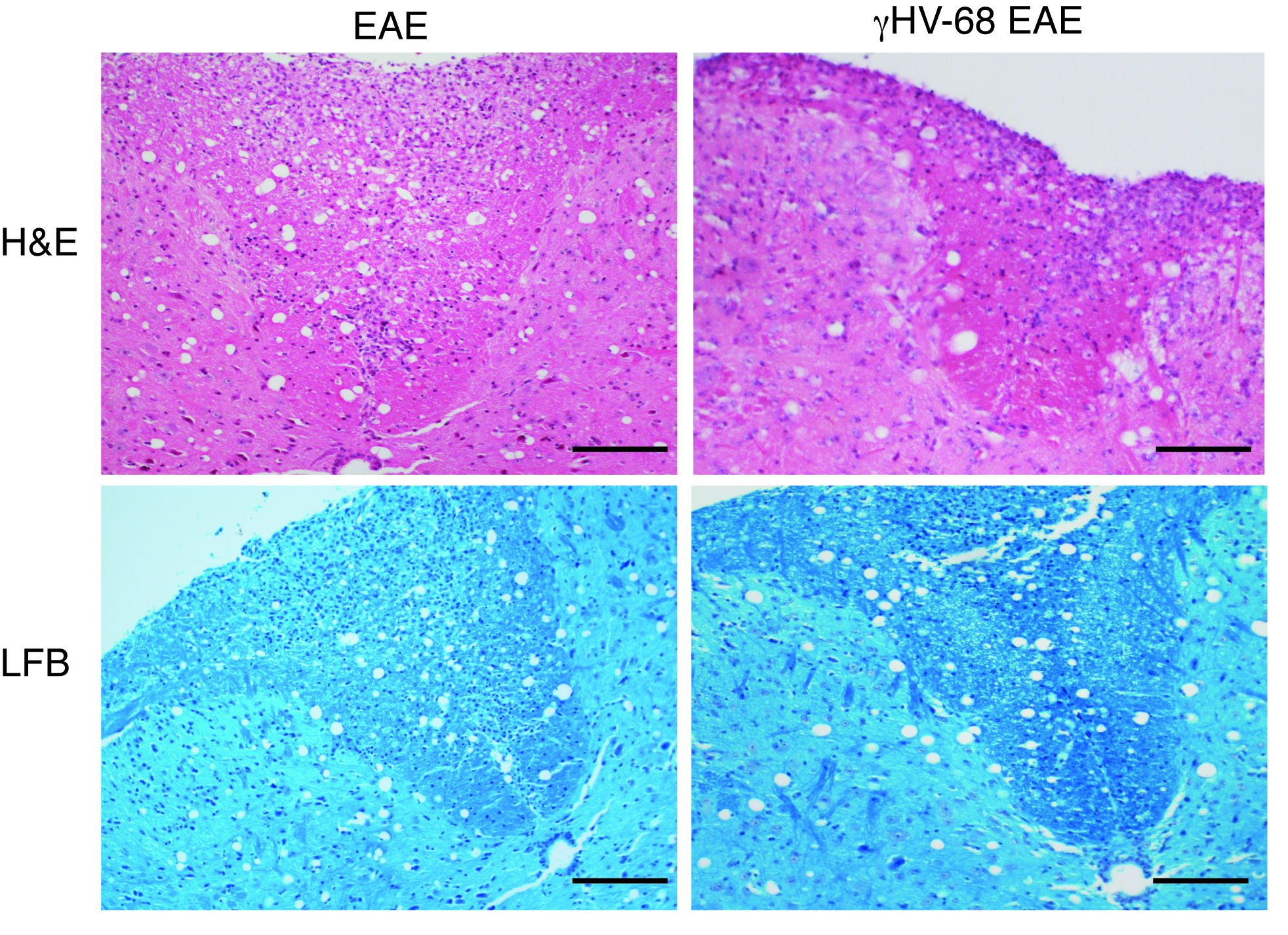

Supplement: Figure S1 — γHV-68 EAE mice show increased amount of immune cells infiltrations in the spinal cords. Mice were infected with γHV-68 (right panels) or MEM only (left panels). Five weeks p.i. EAE was induced. At day 28 post EAE induction mice were perfused and spinal cords were harvested, fixed in formalin and paraffin embedded (similar results obtained at day 15 post EAE induction). Cross-sections were stained with H&E (upper panels) and luxol fast blue (lower panels). Representative pictures of three separate experiments. Scale bar = 100 µm. (TIF) [file ppat.1002715.s001.tif]

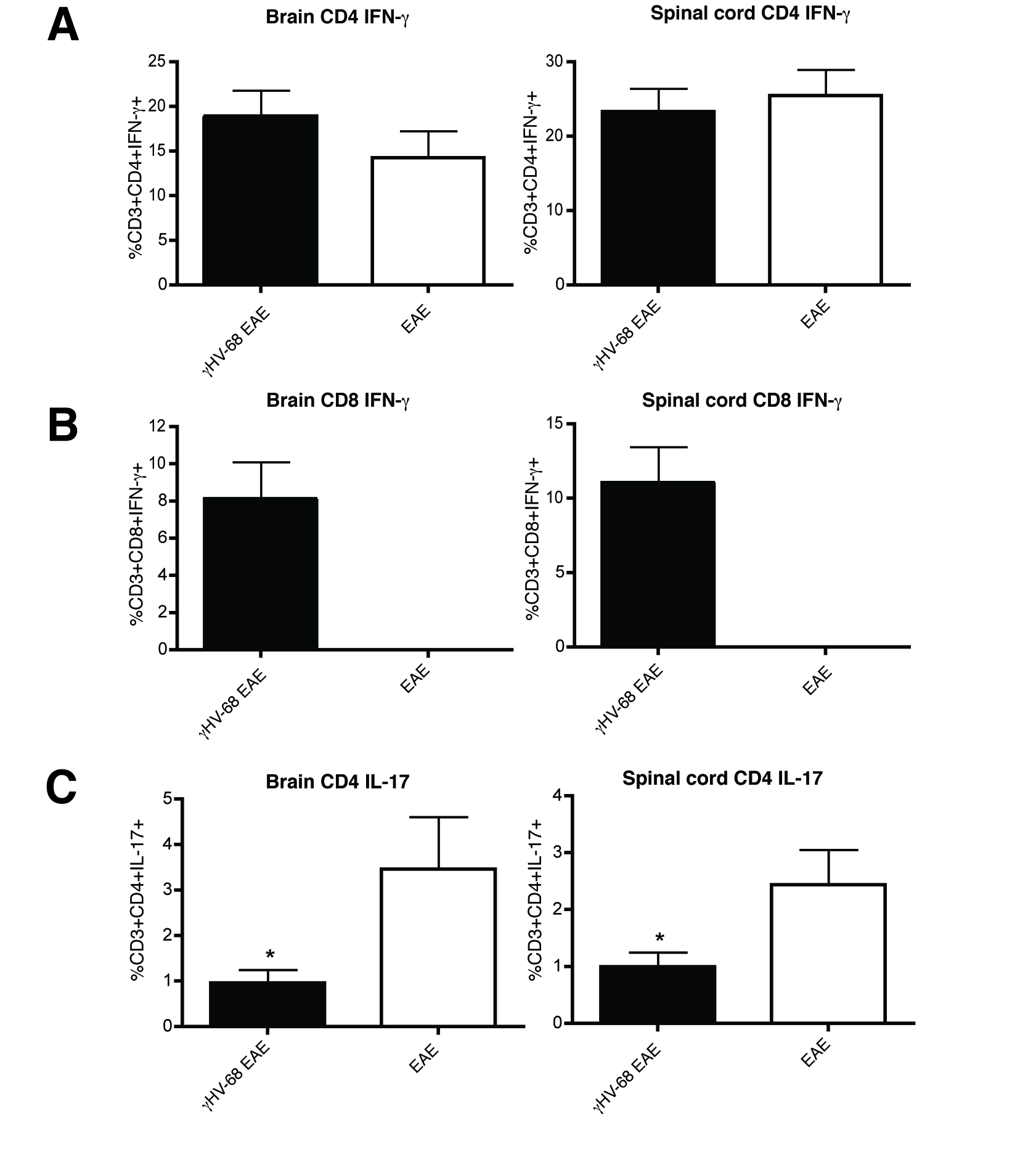

Supplement: Figure S2 — γHV-68 EAE mice show increased T cell expression of IFN-γ accompanied by IL-17 suppression after MOG restimulation. Mice were infected with γHV-68 (black bars, shaded histograms) or MEM only (open bars, open histograms). Five weeks p.i. EAE was induced. At day 14–16 post EAE induction (mean clinical score of 3 for γHV-68 EAE mice, EAE mice were harvested at the same time) mice were perfused, brains (left panels) and spinal cords (right panels) were harvested and processed to isolate immune infiltrates that were restimulated for 24 hours with 100 µM MOG peptide before performing FACS intra cellular staining (A) Percentages of infiltrating CD3+ CD4+ IFN-γ+ lymphocytes. (B) Percentage of infiltrating CD3+ CD8+ IFN-γ lymphocytes (EAE CD8 infiltrations were not enough to perform FACS). (C) CD3+ CD4+ IL-17+ lymphocytes. Two experiments with 6 mice/group. Data were analyzed with t-test: * p<0.05. (TIF) [file ppat.1002715.s002.tif]

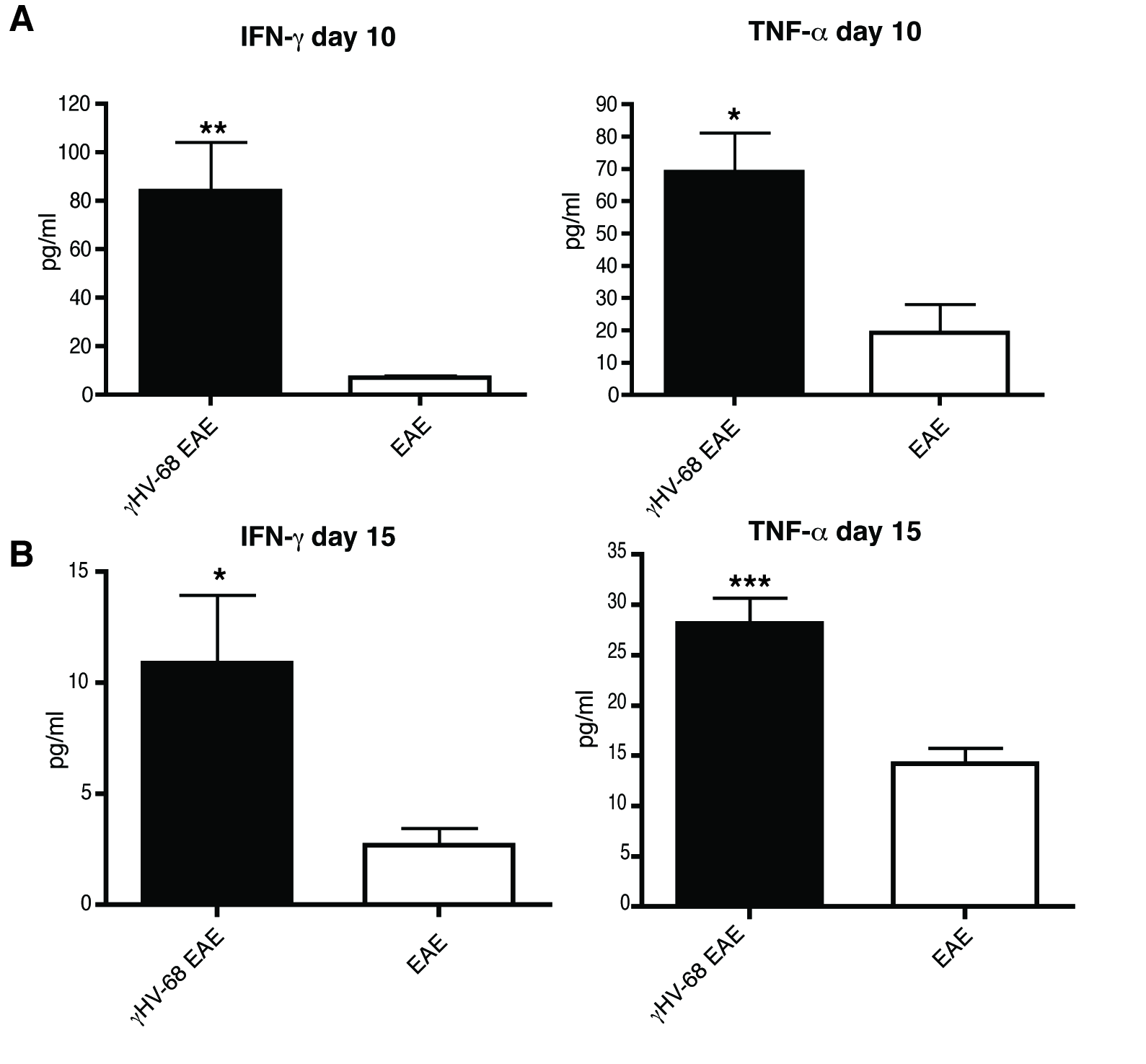

Supplement: Figure S3 — γHV-68 EAE mice show increased levels of pro-inflammatory cytokines in the serum. Mice were infected i.p. with γHV-68 (black bars) or MEM only (open bars). Five weeks p.i., EAE was induced. At day 10 (A) and 15 (B) post EAE induction blood was harvested through a cardiac puncture and the levels of cytokines were evaluated using BD Cytometric Bead Array kits. Three-two separate experiment for each time point with 3–6 mice/group. Data were analyzed with t-test: *** p<0.001; ** p<0.01, * p<0.05. (TIF) [file ppat.1002715.s003.tif]

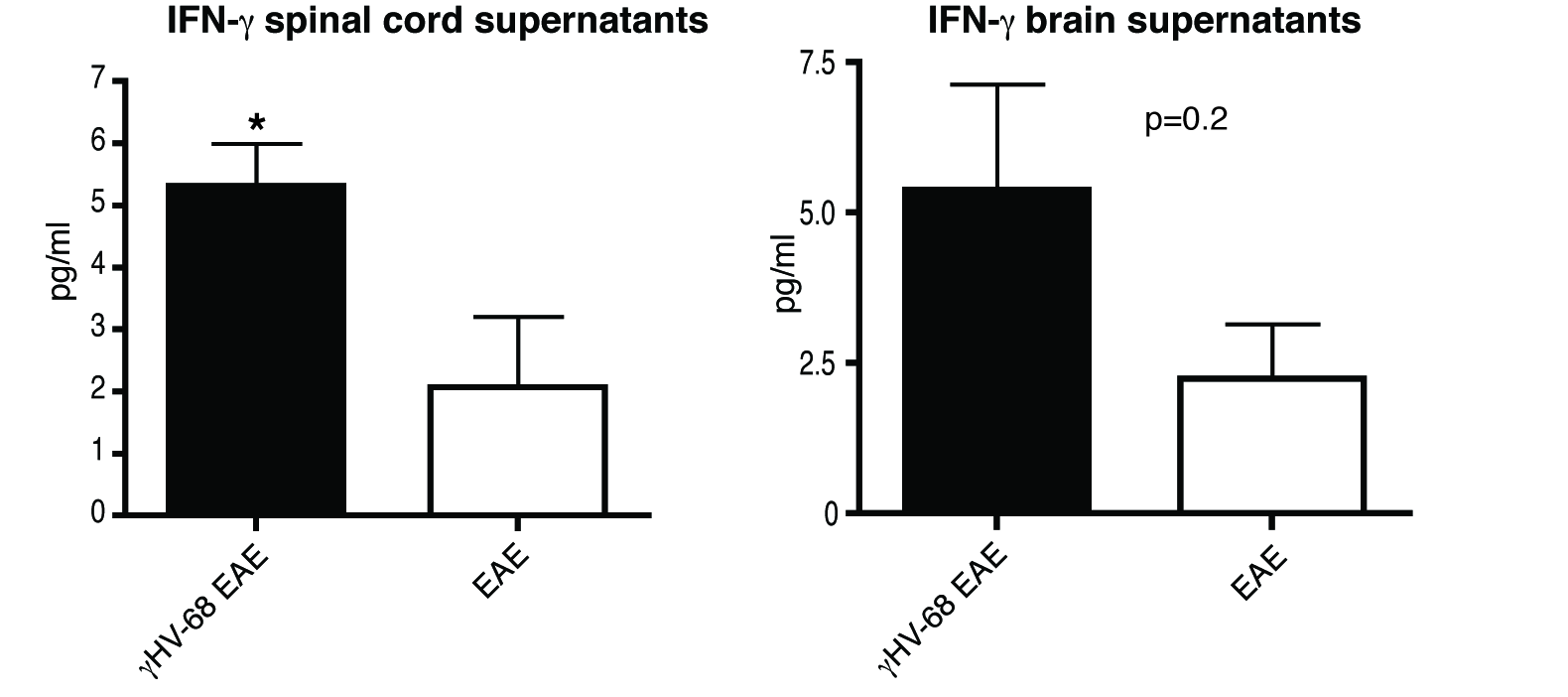

Supplement: Figure S4 — γHV-68 EAE mice show increased levels of IFN-γ in CNS supernatants. Mice were infected i.p. with γHV-68 (black bars) or MEM only (open bars). Five weeks p.i., EAE was induced. At day 14–16 post EAE induction (mean score of 3 for γHV-68 EAE mice, EAE mice were harvested at the same time), mice were perfused and brains and spinal cords were homogenized and the supernatants were analyzed for the presence of cytokines. Levels of cytokines were evaluated using BD Cytometric Bead Array kits. Two separate experiments with 3–6 mice/group. Data were analyzed with t-test: * p<0.05. (TIF) [file ppat.1002715.s004.tif]

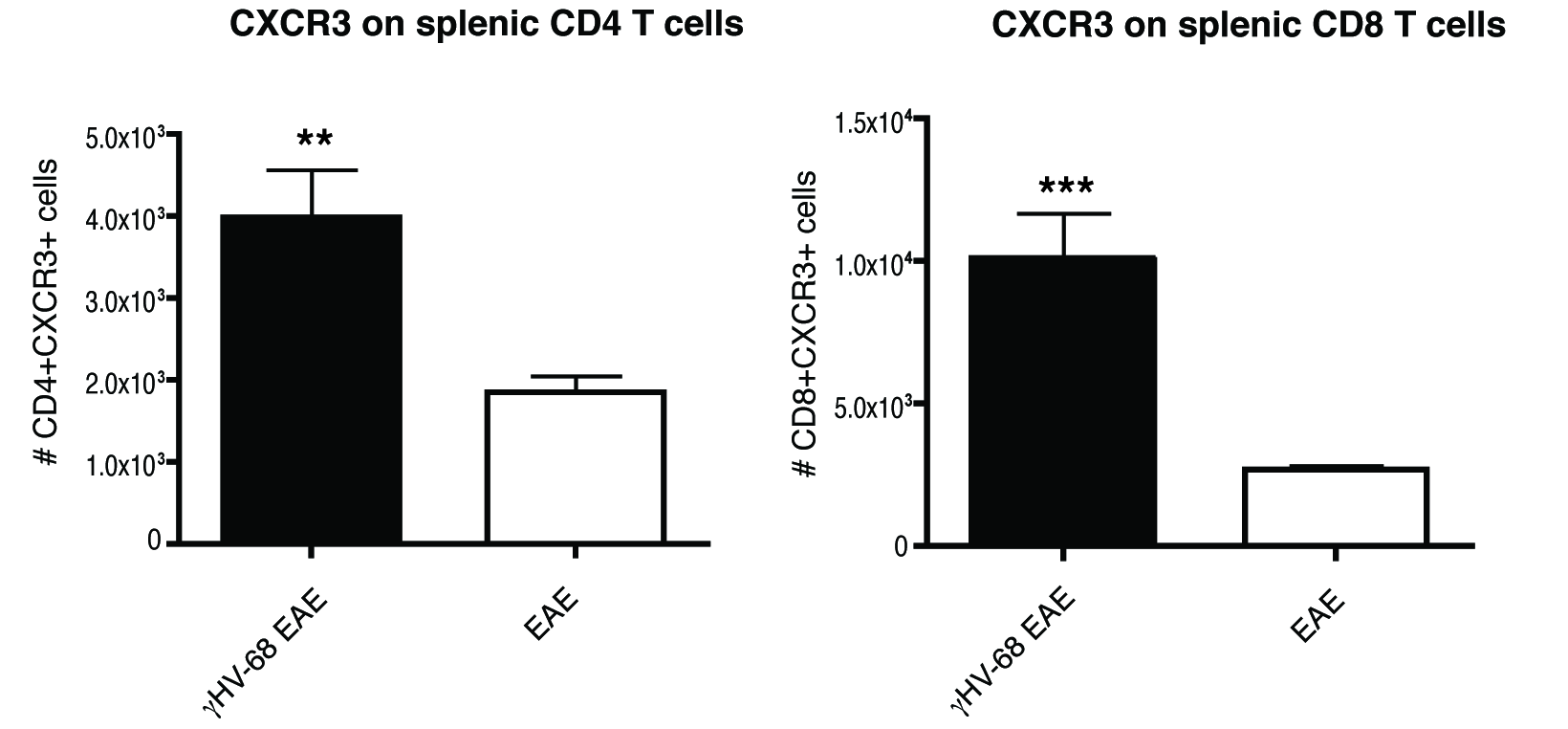

Supplement: Figure S5 — γHV-68 EAE mice show increased levels of CXCR3 on splenic T cells. Mice were infected i.p. with γHV-68 (black bars) or MEM only (open bars). Five weeks p.i., EAE was induced. At day 15 post EAE induction, spleens were harvested and the levels of CXCR3 were assessed through FACS analysis. The histograms show the numbers of CD4+CXCR3+ cells (left panel) or CD8+CXCR3+ cells (right panel) One experiment with 5–6 mice/group. Data were analyzed with t-test: *** p<0.001; ** p<0.01. (TIF) [file ppat.1002715.s005.tif]
